# The effects of reproductive variables on child mortality in Ethiopia: evidence from demographic and health surveys from 2000 to 2016

**DOI:** 10.1186/s12978-023-01734-5

**Published:** 2024-01-10

**Authors:** Hailu Refera Debere, Visseho Adjiwanou

**Affiliations:** 1 Addis Ababa University, Addis Ababa, Ethiopia; 2 Peking University, Beijing, China; 3 University of Quebec in Montreal, Montreal, Canada; 4 University of Montreal, Montreal, Canada

**Keywords:** Child mortality, Conceptual framework, Reproductive variables, Hazard model

## Abstract

**Background:**

Child mortality is a crucial indicator reflecting a country's health and socioeconomic status. Despite significant global improvements in reducing early childhood deaths, Southern Asia and sub-Saharan Africa still bear the highest burden of newborn mortality. Ethiopia is one of five countries that account for half of new-born deaths worldwide.

**Methods:**

This study examined the relationship between specific reproductive factors and under-five mortality in Ethiopia. A discrete-time survival model was applied to analyze data collected from four Ethiopian Demographic and Health Surveys (EDHS) conducted between 2000 and 2016. The study focused on investigating the individual and combined effects of three factors: preceding birth interval, maternal age at childbirth, and birth order, on child mortality.

**Results:**

The study found that lengthening the preceding birth interval to 18–23, 24–35, 36–47, or 48+ months reduced the risk of under-five deaths by 30%, 46%, 56%, and 60% respectively, compared to very short birth intervals (less than 18 months). Giving birth between the ages 20–34 and 35+ reduced the risk by 34% and 8% respectively, compared to giving birth below the age of 20. The risk of under-five death was higher for a 7th-born child by 17% compared to a 2nd or 3rd-born child. The combined effect analysis showed that higher birth order at a young maternal age increased the risk. In comparison, lower birth order in older maternal age groups was associated with higher risk. Lastly, very short birth intervals posed a greater risk for children with higher birth orders.

**Conclusion:**

Not only does one reproductive health variable negatively affect child survival, but their combination has the strongest effect. It is therefore recommended that policies in Ethiopia should address short birth intervals, young age of childbearing, and order of birth through an integrated strategy.

## Background

Child mortality levels and trends serve as important indicators of a country's health and socioeconomic status. Globally, there has been significant progress in reducing child mortality over the past three decades. Ethiopia has actively worked towards reducing child mortality by aligning with international goals and implementing a national plan, resulting in notable successes [1]. A recent official country report revealed that under-five mortality declined from 166 to 67 deaths per 1000 births from 2000 to 2016. However, Ethiopia is still one of the five countries that accounts for fifty percent (50%) of all newborn deaths in the world [2] and still experience high child mortality, with 48 and 67 deaths of infants and under-five per 1000 births, respectively (ECSA, 2016).

This study aims to investigate the impact of risk factors on child survival, with a specific focus on three reproductive factors in Ethiopia: preceding birth intervals, maternal age at childbirth, and birth order. There has been limited attention given to the effects of the combination of these factors on the survival of a child [3].

Extensive research has been conducted on the effects of birth spacing, consistently demonstrating that a short birth interval is associated with a higher risk of under-five mortality. This effect has remained significant even after controlling for the influence of other potential factors [3–15]. The studies revealed that the negative effect of short birth intervals attributed to maternal nutritional depletion, the likelihood of care-seeking behavior, infection transmission, and sibling competition [16–18]. Some studies revealed very long birth intervals have also negative effects [3, 12, 19] associated with increased risks of maternal mortality, pre-eclampsia, eclampsia, and gestational diabetes [19, 20]. However, a very short birth interval has worse consequences than a very long interval [3, 21–24]. Most previous studies consistently revealed the negative effect of young maternal age on children’s survival. Some authors attribute this to the socioeconomic disadvantages of young women and other risk factors, such as income, education, social support, and nutrition factors [25, 26]. They are also associated with higher risks of eclampsia, puerperal Endometriosis, and systemic infections [27] and physiological and anatomical factors associated with young maternal age [28]. Furthermore, some studies have found that births from very young and older women present a higher risk of death compared to births in middle age. Notably, the risk among very young mothers tends to be relatively higher than that of older mothers [7, 15, 24, 29]. The effect of birth order has been also the subject of investigation in various studies, yielding mixed findings and inconsistent results [22, 30]. Studies consistently show that firstborn children face a higher risk of death within the first five years compared to subsequent births [5–7, 9, 13, 23, 31]. A study by Dorothy (1974), cited by Hobcraft, McDonald, and Rutstein (1983), examined the combined effects of age and parity on infant and early childhood mortality risk. The study found that first-order births with maternal age below 25 had a lower risk of death in the first year, while first-order births with maternal age of 30 and above had a higher risk. Additionally, births of third and fourth (3–4) birth orders with mothers below the age of 25 had a significantly higher risk of infant mortality. According to the Ethiopian Mini Demographic and Health Survey of 2019, the mean age at first birth of women aged 25–49 is 18.7 years. The study indicated that median age at first birth in Ethiopia varies by religion 21 years for Orthodox Christians and 19 for Muslims [32].

The study also indicated that, apart from the first birth, variations in mortality risk could be attributed to birth spacing rather than age and birth order [33]. Rutstein maintains that the risk of infant mortality associated with different preceding birth intervals outweighs the risk attributed to maternal ages or birth orders. This suggests that maternal age at childbirth and birth order alone cannot fully represent the influence of preceding birth intervals on early childhood mortality. The study conducted in Sri Lanka supports this conclusion on the effect of the preceding birth interval in the presence of maternal age at childbirth and birth order [15]. The study also revealed that a long birth interval is more advantageous for higher birth order births [34].

A recent study explored the combined effects of three reproductive variables [35]. The findings indicate that first-born children with a maternal age at birth below 18 and short birth intervals have the highest mortality risk. However, delaying the first birth until ages above 23 increases the risk for both the first child and older siblings. Children of higher birth order with very young mothers also face elevated mortality. In contrast, children of second and third birth order (2–3) with older mothers have the lowest mortality rates.

The conceptualization of this study on child mortality in Ethiopia is based on a thorough review of existing literature and considering two prominent theoretical frameworks. The first framework employed is Mosley and Chen's model, which provides a comprehensive understanding of the factors influencing child mortality. The second framework, proposed by Sastry, offers additional insights into the context-specific determinants of child mortality [36]. By referring to these frameworks, this study aims to identify and examine the covariates associated with child mortality in Ethiopia, contributing to a deeper understanding of this critical issue. The former is designed to study the determinants of child mortality in developing countries in general [37], whereas the latter has built its conceptualization on the former, mainly focusing on classifying the factors at different levels (individual level, household level, and community level). Most of the previous studies focused on separate effects of the reproductive variables (preceding birth intervals, maternal age, and birth order). However, this study aimed and extended to explore the combined effects of these factors in addition to the separate effects.

## Data and methods

This study is based on Ethiopian Demographic and Health Survey (DHS) data from 2000, 2005, 2011 and 2016. This study considered all single births in the last 5 years preceding each survey. However, for this reason, some important characteristics that explain the health status of the mother and birth during pregnancy and after delivery were collected only for the last birth, and the study was limited to only recent births to the women in the last 5 years preceding each survey. Furthermore, since we do not have a preceding birth interval for the first birth, we conducted the analysis in the model separately for a first birth. Overall, the study considered 30,411 samples of births, which summarizes 7890, 7237, 7814, and 7470 births from the 2000, 2005, 2011 and 2016 Ethiopian demographic and health survey data, respectively. The data of all four surveys were pooled together for the multivariate analysis.

### Variables of the study

The dependent variable (survival time) was broken down into months clustered into the wider discrete form of five categories: 0, 1–5, 6–11, 12–23, and 24–59, similar to previous studies [38, 39].

The main independent variables of this study are birth spacing, maternal age at childbirth, and birth order. The covariates, which include the main independent and other important factors are identified by referring to a framework of determinants of child mortality and the literature.

### Model

The hazard rate is the quantity used to investigate the risk of event occurrence at each discrete time point. Suppose ${\text{T}}$ denotes a discrete-time random variable and ${\text{t}}$ is the specified value of ${\text{T}}$. The discrete-time hazard function represented by ${\text{h}}({\text{t}})$ is the probability that an event happened at time ${\text{t}}$, given that an individual survived to an earlier time; it is given as:

${\text{h}}\left({\text{t}}\right)={\text{P}}({\text{T}}={\text{t}}|{\text{T}}\ge {\text{t}})$ more specifically, this can be defined as

$${\text{h}}\left({\text{t}}\right)=\frac{\mathrm{Number \, of \, events \, at \, discrete}-\mathrm{time \, t}}{\mathrm{Number \, at \, risk \, at \, discrete}-\mathrm{time \, t}}$$

The magnitude of the hazard function shows the risk of occurrence of an event, and as a probability, its values range between 0 and 1.

### Discrete-time hazard function model

After data are restructured, logit transformation of the hazard function is commonly used in modeling the association with predictors. Given that, the discrete-time hazard function is formulated as

$$h_{ti} = \Pr (y_{ti} = 1|y_{t - 1,i} = 0)$$

_where_

$$y_{ti} = \left\{ {\begin{array}{*{20}c} {1,} & {if\;ai^{the} \;is\;child\;is\;died\;at\;time\;t} \\ {0,} & {f\;ai^{the} \;is\;child\;is\;alive\;at\;time\;t} \\ \end{array} } \right.$$

The discrete-time logit model is defined as

$$\mathrm{logit }\mathbf{h}\left(\mathbf{t}\right)={\varvec{\upalpha}}\left(\mathbf{t}\right)+{\varvec{\upbeta}}\mathbf{X}$$

where** α** denotes the intercept and **β** represents the set coefficients of the predictors.

Specifically, suppose the hazard of a discrete-time period ${\text{j}}$ for individual ${\text{i}}$ is represented by ${\text{h}}\left({{\text{t}}}_{{\text{ij}}}\right)$, the fitted logit of this hazard in this specified period for the selected individual is associated with ${\text{P}}$ predictors as:

$$\mathrm{logit h}\left({{\text{t}}}_{{\text{ij}}}\right)=\left[{\mathrm{\alpha }}_{1}{{\text{D}}}_{1{\text{ij}}}+{\mathrm{\alpha }}_{2}{{\text{D}}}_{2{\text{ij}}}+\dots +{\mathrm{\alpha }}_{{\text{K}}}{{\text{D}}}_{{\text{Kik}}}\right]+[{\upbeta }_{1}{{\text{X}}}_{1{\text{ij}}}+{\upbeta }_{2}{{\text{X}}}_{2{\text{ij}}}+\dots +{\mathrm{\alpha \beta }}_{{\text{P}}}{{\text{X}}}_{{\text{Pik}}}]$$

This model has two parameters, which are vectors ${\varvec{\upalpha}}$ and ${\varvec{\upbeta}}$**.** The intercepts ${\mathrm{\alpha }}_{1}, {\mathrm{\alpha }}_{2}, \dots , {\mathrm{\alpha }}_{{\text{K}}}$ are the baseline logit of hazard in each period. The second set of parameters ${\upbeta }_{1},{\upbeta }_{2}, \dots , {\upbeta }_{{\text{P}}}$, which are coefficients of predictors, give the effect of a unit increase in a predictor, holding other predictors constant. Intercept ${\mathrm{\alpha }}_{{\text{t}}}$ is a logit baseline hazard function, while ${{\text{x}}}_{{\text{ti}}}$ is a covariate, whether constant over time or time-varying, and β vector of coefficients of the covariates.

Transforming the logit representation model of the hazard function to predictors can be given as:

$${\text{h}}\left({{\text{t}}}_{{\text{ij}}}\right)= \frac{1}{1+{{\text{e}}}^{-\left(\left[{\mathrm{\alpha }}_{1}{{\text{D}}}_{1{\text{ij}}}+{\mathrm{\alpha }}_{2}{{\text{D}}}_{2{\text{ij}}}+\dots +{\mathrm{\alpha }}_{{\text{K}}}{{\text{D}}}_{{\text{Kik}}}\right]+[{\upbeta }_{1}{{\text{X}}}_{1{\text{ij}}}+{\upbeta }_{2}{{\text{X}}}_{2{\text{ij}}}+\dots +{\mathrm{\alpha \beta }}_{{\text{P}}}{{\text{X}}}_{{\text{Pik}}}]\right)}}$$

Maximum likelihood derives the estimates of the parameters ${\varvec{\upalpha}}$ and ${\varvec{\upbeta}}$**,** which maximize the likelihood of observing the sample data. The likelihood function for the discrete function hazard model is defined as:

$${\text{Likelihood}} = \prod\limits_{i = 1}^{n} {\prod\limits_{j = 1}^{Ki} {h(t_{ij} )^{{Event_{ij} }} (1 - h(t_{ij} ))^{{(1 - Event_{ij} )}} } }$$

where $h(t_{ij} )$ is the probability that an individual experiences an event of interest in period ${\text{j}}$ and $(1-{\text{h}}({{{\text{t}}}_{{\text{i}}}}_{{\text{j}}}))$ is the probability that an individual does not experience the event of interest in period${\text{j}}$.

Based on the maximum likelihood function, we apply different alternative models using figures of likelihood and deviance statistics, AIC, and BIC.

The model:

$$\mathrm{logit h}\left({{\text{t}}}_{{\text{ij}}}\right)=\left[{\mathrm{\alpha }}_{1}{{\text{D}}}_{1{\text{ij}}}+{\mathrm{\alpha }}_{2}{{\text{D}}}_{2{\text{ij}}}+\dots +{\mathrm{\alpha }}_{{\text{K}}}{{\text{D}}}_{{\text{Kik}}}\right]+\left[{\upbeta }_{1}{{\text{X}}}_{1{\text{ij}}}+{\upbeta }_{2}{{\text{X}}}_{2{\text{ij}}}+\dots +{\mathrm{\alpha \beta }}_{{\text{P}}}{{\text{X}}}_{{\text{Pik}}}\right]+ [{\upgamma }_{1}{{\text{Z}}}_{1{\text{ij}}}+{\upgamma }_{2}{{\text{Z}}}_{2{\text{ij}}}+\dots +{\upgamma }_{{\text{Q}}}{{\text{Z}}}_{{\text{Qik}}}]$$

The reduced model:

$$\mathrm{logit h}\left({{\text{t}}}_{{\text{ij}}}\right)=\left[{\mathrm{\alpha }}_{1}{{\text{D}}}_{1{\text{ij}}}+{\mathrm{\alpha }}_{2}{{\text{D}}}_{2{\text{ij}}}+\dots +{\mathrm{\alpha }}_{{\text{K}}}{{\text{D}}}_{{\text{Kik}}}\right]+[{\upbeta }_{1}{{\text{X}}}_{1{\text{ij}}}+{\upbeta }_{2}{{\text{X}}}_{2{\text{ij}}}+\dots +{\mathrm{\alpha \beta }}_{{\text{P}}}{{\text{X}}}_{{\text{Pik}}}]$$

To assess whether that there is a statistical improvement in fitting the model by adding predictors, ${{\text{Z}}}_{1}$, ${{\text{Z}}}_{2}$, …, ${{\text{Z}}}_{{\text{k}}}$ to the model, we test the combined hypothesis as H0: ${\upgamma }_{1}={\upgamma }_{2}=\dots ={\upgamma }_{{\text{Q}}}=0$ by comparing values of the deviance statistic. In comparing two models that are not nested in one another, we use BIC: the model with the smaller BIC value is considered the better model.

### Modeling the effects of reproductive variables on childhood survival

To assess the influence of the three reproductive factors (preceding birth interval, maternal age, and birth order), we fitted different models, from the null model to the additive model of the three main independent variables to the multiplicative model (or interaction model) and compare the goodness of fit of alternative models consecutively to decide which predictors should be included and which should be left aside, as presented in Table 1. We graphically presented the predicted risk of the combined effect of the reproductive variable based on multiplicative models. The list of other independent variables included in the models has been given in Table 2 in the result section with summary statistics. Accordingly, model_4 is the final model from the additive model which includes all three main independent variables and other independent variables together mentioned in Table 2 in the result sections and all the three multiplicative models (Model _ 5a, Model _ 5b, and Model _ 5c) have been considered and the risk of mortality with the combined effects of the three reproductive variables presented graphically.

**Table 1:** Models fitted to assess the effects of the reproductive variables

Models		Main independent variables				
		Birth interval	Maternal age	Birth order	Other independent variables	Deviance
Null						
	Model_0					12,079
Additive						
	Model _ 1a	X				12,003
	Model _ 1b		X			12,030
	Model _ 1c			X		12,049
	Model _ 2a	X			X	11,489
	Model _ 2b		X		X	11,531
	Model _ 2c			X	X	11,550
	Model _ 3a	X	X		X	11,450
	Model _ 3b	X		X	X	11,472
	Model _ 3c		X	X	X	11,524
	Model _ 4	X	X	X	X	11,447
Multiplicative						
	Model _ 5a	X	X		X	11,440
	Model _ 5b	X		X	X	11,439
	Model _ 5c		X	X	X	11,443

**Table 2:** Number of births and percent distributions of deaths by covariates and years

	EDHS_2000		EDHS_2005		EDHS_2011		EDHS_2016	
	Births (N)	Died (%)	Births (N)	Died (%)	Births (N)	Died (%)	Births (N)	Died (%)
*Birth interval (months)*								
First birth	1362	11.45	1190	8.99	1399	4.72	1434	2.65
< 18	423	10.17	410	12.44	414	5.80	418	8.37
18–23	629	9.38	663	7.54	598	4.68	645	5.43
24–35	2410	6.76	1991	5.47	2201	4.95	1878	3.78
36–47	1667	5.58	1535	4.69	1600	4.38	1235	3.08
48 +	1399	6.08	1448	4.14	1602	3.37	1860	2.85
*Birth order*								
1	1362	11.45	1190	8.99	1399	4.72	1434	2.65
2–3	2341	6.41	2082	5.04	2434	3.57	2247	3.47
4–6	2444	5.69	2365	4.82	2477	4.93	2336	3.00
7 +	1743	8.78	1600	7.75	1503	4.99	1452	5.79
*Maternal age at birth (years)*								
< = 19	1011	9.79	992	10.18	945	5.19	835	3.95
20–34	5247	6.63	4884	5.12	5576	4.04	5340	3.18
35 +	1632	9.38	1361	7.27	1292	5.96	1295	5.10
*Age at status at event or censor (months)*								
0	332	66.87	324	63.89	298	66.44	239	55.23
1–5	1074	11.55	1139	10.18	1208	5.05	1030	4.47
6–11	1205	9.96	1101	4.63	1137	2.99	1070	3.27
12–23	2150	4.05	1831	2.29	1863	1.66	1956	1.74
24–59	3129	1.50	2842	1.20	3306	0.82	3159	0.73
*Wealth index*								
Poorest	1626	4.61	1501	5.46	1719	5.64	1632	3.92
Poorer	1665	6.73	1540	6.49	1681	4.88	1632	3.37
Middle	1652	9.93	1565	7.67	1619	3.77	1564	3.64
Richer	1598	8.45	1440	5.69	1459	3.77	1389	4.18
Richest	1349	8.38	1191	5.54	1335	4.27	1252	2.80
*Maternal education*								
No education	6488	8.09	5673	6.43	5214	4.56	4718	4.03
Primary	983	5.60	1199	6.26	2239	4.69	2117	3.16
Secondary and above	418	4.78	367	3.00	361	2.22	635	2.05
*Residence*								
Urban	897	8.36	631	5.71	1174	4.51	950	2.32
Rural	6993	7.51	6607	6.27	6640	4.49	6520	3.79
*Child wanted*								
Then	4748	8.11	4551	7.01	5296	5.06	5488	3.41
Later	1544	4.66	1334	3.90	1661	2.65	1312	3.43
No more	1594	8.85	1345	5.58	854	4.57	670	5.67
*Child sex*								
Male	4066	7.77	3738	6.90	4031	5.53	3869	4.68
Female	3824	7.43	3500	5.49	3783	3.38	3601	2.44
*Tetanus*								
0	5740	8.22	4487	7.24	4146	4.80	3344	4.67
1–3	1890	5.87	2372	4.51	3294	4.31	3569	2.83
4 +	184	4.89	300	4.00	254	2.76	375	2.93
*Place of delivery*								
Health facility	425	7.06	458	7.64	918	5.77	2355	2.51
Home	7446	7.60	6745	6.09	6863	4.34	5003	4.12
Others	18	11.11	29	3.45	29	0.00	112	4.46
*ANC (antenatal care)*								
0	5730	7.80	5184	6.37	4457	4.64	2775	5.23
1–3	1296	8.02	1143	5.69	1835	4.74	2308	3.21
4 +	816	4.66	880	5.68	1495	3.81	2372	2.11
*Water*								
Improved source	1248	8.01	4162	6.22	3657	3.88	4359	4.04
Others or not dejure	307	9.12	82	7.32	259	3.86	135	0.00
Unimproved source	6334	7.44	2993	6.18	3892	5.11	2976	3.13
*Sanitation*								
Improved facility	36	16.67	682	6.01	1032	3.00	818	2.08
Others or not dejure	302	8.61	70	5.71	238	2.10	150	1.33
Unimproved facility	7551	7.51	6486	6.24	6543	4.81	6502	3.86
*Mode of delivery*								
Caesarian section	56	8.93	84	9.52	142	7.75	171	8.77
Non-caesarian section	7824	7.59	7151	6.18	7671	4.43	7299	3.49
*SBA (skilled birth assistance)*								
No	7068	7.72	7226	6.19	6209	4.41	5004	4.06
Yes	820	6.34	5	0.00	1600	4.81	2466	2.68
*Region*								
Tigray	532	6.02	478	4.60	520	3.85	528	2.08
Afar	84	10.71	68	10.29	77	6.49	70	5.71
Amhara	2204	7.30	1843	7.92	1978	4.04	1605	3.24
Oromia	3024	7.84	2696	5.75	3077	4.78	3081	4.25
Somali	83	6.02	283	7.42	194	4.12	264	4.55
Benishangul	80	6.25	68	5.88	91	4.40	80	2.50
SNNP	1672	8.13	1614	5.45	1611	5.03	1577	3.23
Gambela	22	9.09	23	4.35	30	3.33	20	5.00
Harari	16	6.25	15	0.00	19	5.26	17	5.88
Addis Ababa	146	6.16	126	3.17	190	2.11	194	1.55
Dire Dawa	26	7.69	24	4.17	26	3.85	33	3.03

## Results and analysis

### Trends of the risk of under-five mortality in Ethiopia

**Fig. 1:**
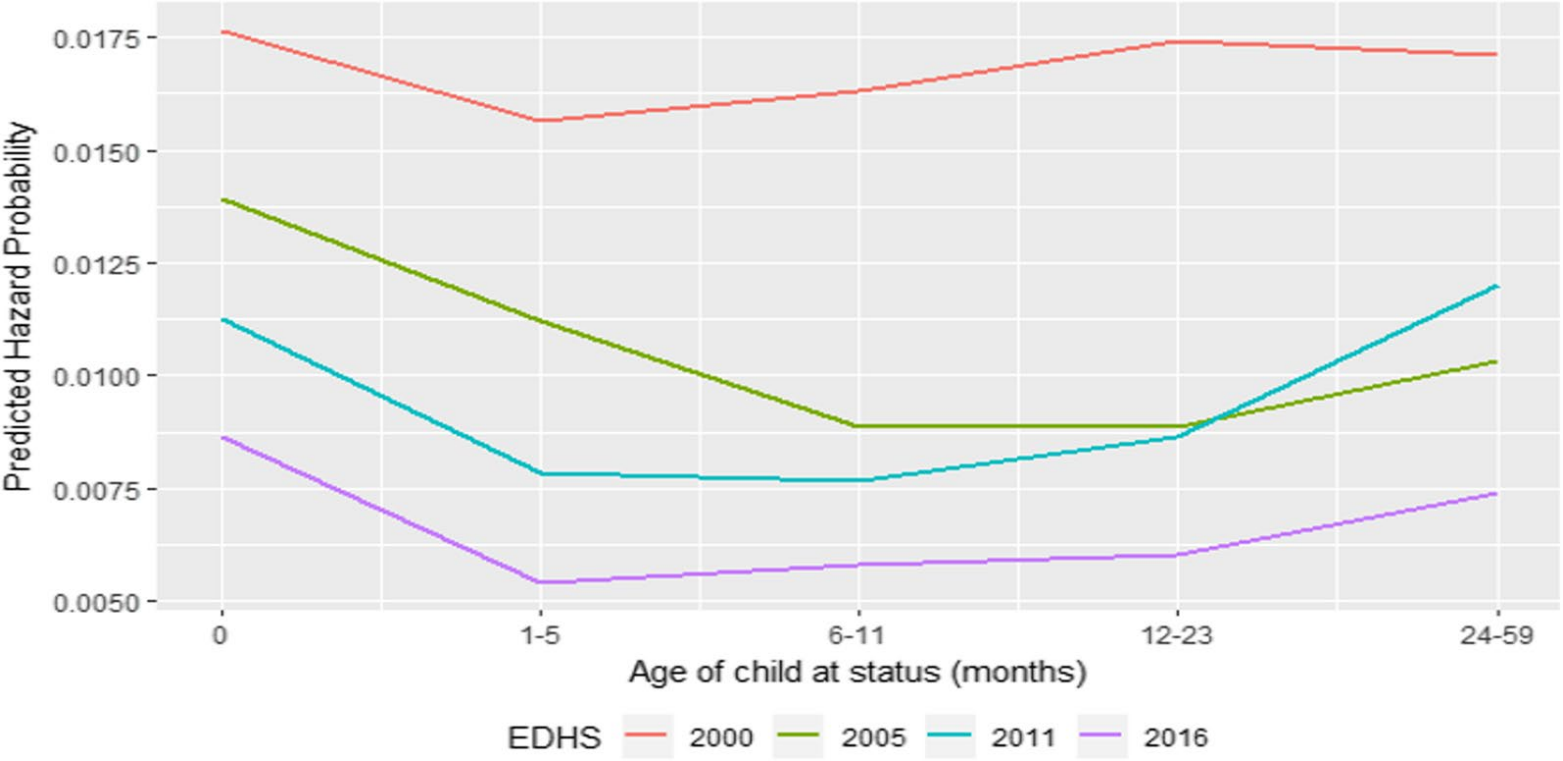
Differentials of under-five mortality by years, Ethiopia

Overall, the risk of child death has decreased over the years across all age groups. The highest risk of death is observed during the neonatal stage. Figure 1 illustrates a decline in risk from the peak during the neonatal period, which remains relatively stable from 6 months to the first year of the child's life. However, the risk of death gradually increases throughout the remaining childhood period.

### Reproductive variables and child mortality: a descriptive assessment

Table 2 provides information on the weighted number of births and the distribution of child deaths across various categories of the three main independent variables and other independent variables, categorized by period. The total births mentioned in each EDHS (Ethiopian Demographic and Health Survey) represent the overall sample size of the study, which consists of approximately 30,411 births. These births encompass all births that occurred within the last five years of the respective surveys, excluding any births that were excluded due to missing observations for certain covariates.

Overall, the table demonstrates that a short birth interval of less than 18 months is associated with the highest proportions of child deaths. Additionally, there is a notable increase in the proportion of under-five deaths for higher birth orders (7 and above) compared to birth orders ranging from 2 to 6. Furthermore, among very young mothers (≤ 19 years) and older mothers (35 years and above), a higher proportion of children experience mortality compared to mothers within the middle age range (20 to 34 years).

### Effects of the reproductive variables

In this section, we present the results of the main effect and combination effect analysis of the reproductive variables. Table 3 presents the results of the four models (3a to 4).

**Table 3:** Main effects model fitted with preceding birth interval and maternal age (model_3a), preceding birth interval and birth order (model_3b), maternal age and birth order (model_3c) and all together (model_4) with control variables

	Model_3a	Model_3b	Model_3c	Model_4
*Age at status (event or censor)*				
0	− 1.93 (0.36)***	− 2.23 (0.34)***	− 2.51 (0.35)***	− 1.91 (0.36)***
1–5	− 2.63 (0.36)***	− 2.94 (0.35)***	− 3.22 (0.35)***	− 2.62 (0.36)***
6–11	− 2.81 (0.36)***	− 3.12 (0.35)***	− 3.40 (0.35)***	− 2.80 (0.36)***
12–23	− 2.80 (0.37)***	− 3.10 (0.35)***	− 3.38 (0.36)***	− 2.79 (0.37)***
24–59	− 2.56 (0.37)***	− 2.87 (0.35)***	− 3.14 (0.36)***	− 2.55 (0.37)***
*Preceding birth intervals*				
18–23	− 0.36 (0.12)**	− 0.36 (0.12)**		− 0.36 (0.12)**
24–35	− 0.61 (0.10)***	− 0.61 (0.10)***		− 0.61 (0.10)***
36–47	− 0.84 (0.11)***	− 0.84 (0.11)***		− 0.83 (0.11)***
48 +	− 0.93 (0.11)***	− 0.90 (0.11)***		− 0.91 (0.11)***
*Maternal age*				
20–35	− 0.35 (0.12)**		− 0.60 (0.13)***	− 0.42 (0.13)**
35 +	0.07 (0.13)		− 0.36 (0.15)*	− 0.08 (0.16)
*Birth order*				
4–6		0.06 (0.07)	0.11 (0.08)	0.09 (0.08)
7 +		0.32 (0.08)***	0.27 (0.10)**	0.19 (0.10) ~

Level of significance: ***p $<$ 0.001, **p $<$ 0.01, *p $<$ 0.05, ~ p $<$ 0.1

The results of the model show that all three reproductive factors (preceding birth interval, maternal age at childbirth and birth order) contribute significantly to under-five mortality in Ethiopia. All the coefficients of categories of birth intervals are negative, and a corresponding odds ratio of child mortality is less than one, which generally suggests that births with short preceding birth intervals (< 18 months) experience a higher risk of death in under-five than any other intervals. Furthermore, the result tells us that holding other factors constant at a fixed value, including maternal age at the child’s birth and birth order, the odds of death for under-five births with intervals 18–23, 24–35, 36–47 and 48 + months are reduced by 30 percent (OR = 0.70), 46 percent (OR = 0.54), 56 percent (OR = 0.44) and 60 percent (OR = 0.40), respectively, compared to births after short birth intervals (< 18 months). Overall, it shows that the increase in birth intervals reduces the risk of child death, with consistently higher intervals leading to a lower risk of under-five deaths in Ethiopia. Furthermore, the results reveal that although higher birth intervals are associated with lower odds of death, the effect of birth intervals decreases with increasing birth intervals.

Maternal age at a child’s birth also significantly affects child survival status. Holding other factors included in the model fixed, the odds of under-five mortality in births with maternal age at the child’s birth of 20–34 and 35 + years decreases by 34 percent (OR = 0.66) and 8 percent (OR = 0.92), respectively, compared to births of maternal age below twenty. The result reveals that maternal age at the child’s birth of 20–34 years is more advantageous for child survival than a younger and older maternal age. Furthermore, the results show that birth orders 2 and 3 are the most advantageous for the survival of under-fives in Ethiopia. The odds of under-five mortality were significantly (p < 0.1) higher in the 7 + than in the 2–3 birth order by 17 percent (OR = 0.0.83). The results also indicate that births with birth orders 4–6 are more likely to die by 8 percent (OR = 0.92) than those with birth orders 2–3.

**Fig. 2:**
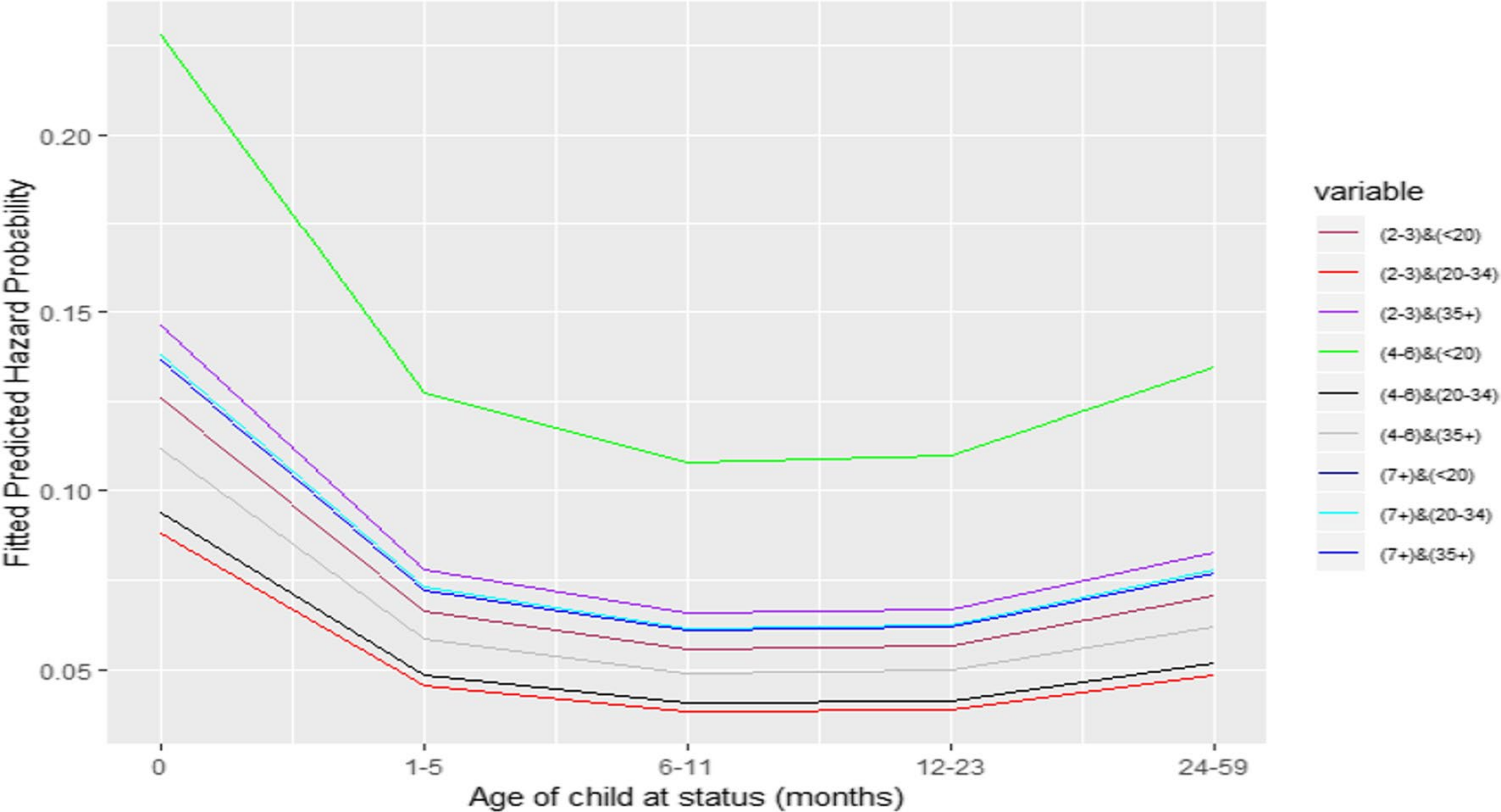
Combined effect of maternal age at childbirth and birth order

**Fig. 3:**
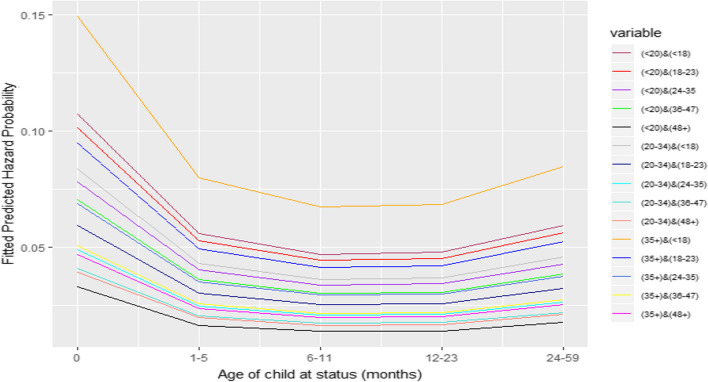
Combined effect of maternal age at childbirth and preceding birth interval

**Fig. 4:**
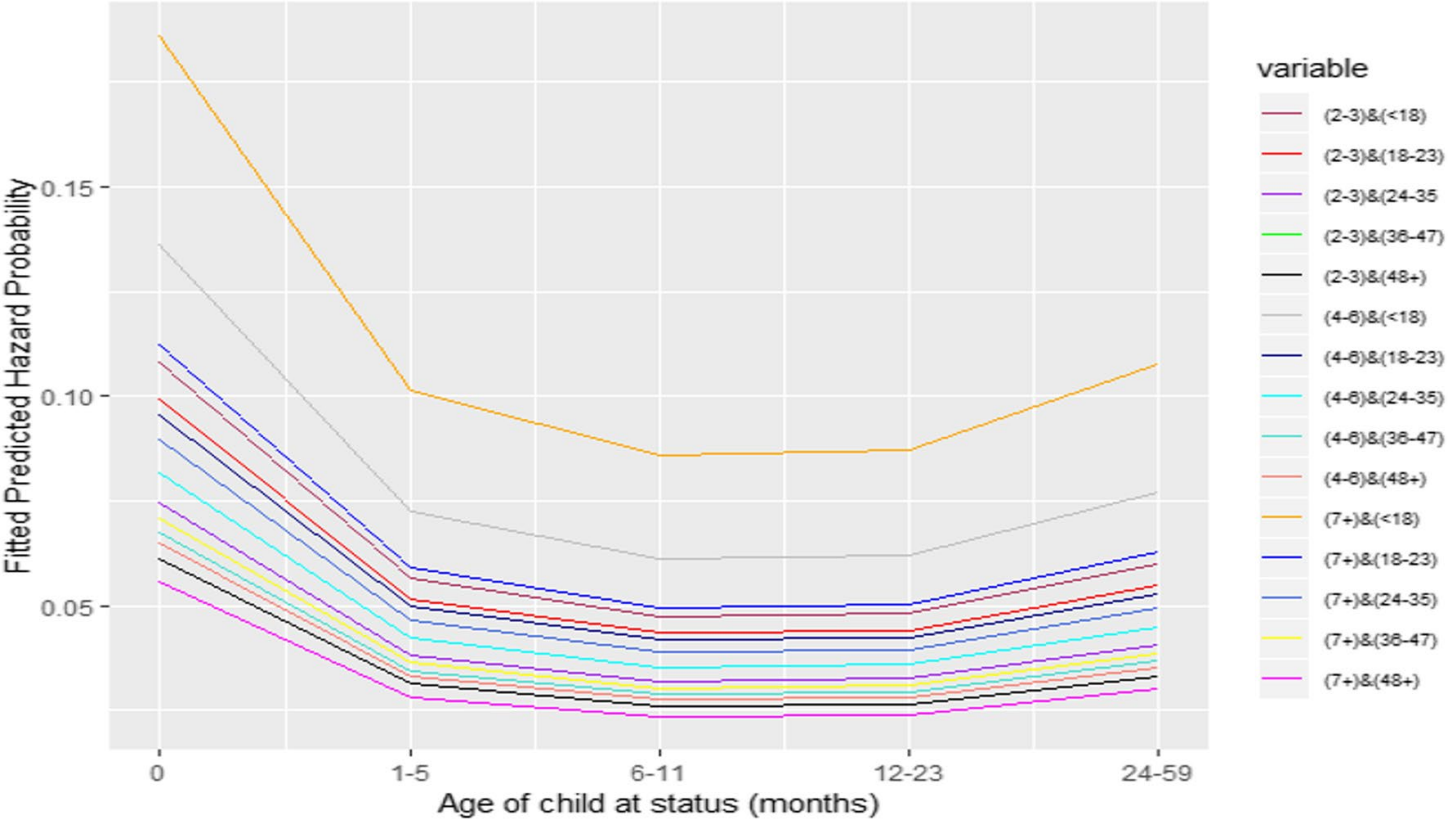
Combination effect of preceding birth interval and birth order

In the next process, we fitted models to examine the combined effect of the three reproductive variables. To facilitate the interpretation of the effects of combinations of the three reproductive factors, the respective predicted hazard probability plots of the under-five mortality rate in Ethiopia are presented below in Figs. 2, 3 and 4.

The results show that holding the main effect of other factors, including the main effect of preceding birth interval fixed, the births with the higher birth order (4–6) and lower maternal age at the child’s birth (< 20) are associated with the highest risk of death, followed by lowest birth order (2–3) in oldest maternal age (35 +). These trends reveal that the higher risk is attributed to the higher birth order in young maternal age at a child’s birth and lower birth order in older maternal age. At older maternal age, the child with the lower birth order (2–3) experiences a higher risk of death than those of higher birth order (4 +). Overall, 2nd and 3rd birth order births to women aged 20–34 at childbirth are advantageous for the survival of under-fives in Ethiopia.

By controlling the main effect of other independent variables and birth order, the effects of the combinations of maternal age at the child’s birth and preceding birth intervals are investigated. It can be noticed that the shortest interval (< 18 months) affects the mortality of children of the oldest mother (35 + years) more than those of very young mothers (< 20 years). In contrast, a very long interval (48 + months) is more advantageous for births of very young mothers (< 20 years) than those of older mothers (35 +). Furthermore, births with preceding birth intervals of over three years among younger mothers (< 35 months) are less likely to be exposed to the risk of mortality of children under-five years of age when compared to those with mothers over age 35 at birth.

Finally, the effects of the combinations of preceding birth intervals and birth order on the survival status of under-fives were assessed and revealed the high effect of a longer birth interval on the survival of births with lower and higher birth orders. Holding constant the effect of other covariates, including the main effect of maternal age at childbirth, it has been noted that a very short birth interval increases the risk of under-five deaths for the higher order births (≥ 4) more than lower birth order births. On the other hand, although a very long birth interval is advantageous for the survival of births of any birth order, it is more so for those of higher birth orders. Generally, a very short interval is associated with a high risk of under-five mortality in both very low and high birth orders, and a very long interval improves the survival of births, more so in the much lower and higher birth orders than in the middle birth order (4–6).

In Table 4, the effect of maternal age at birth of first-borns is assessed. The results show that in Ethiopia, maternal age at birth does not significantly influence the survival of the first-born child. Although it is significant in a reduced model with child age at the 10 percent significance level, its effect is not significant in the presence of other covariates. However, the results indicate that relative to the maternal age at the child’s birth of < 20 years, the risk is higher at older ages. Furthermore, a higher maternal age leads to a higher risk for firstborns in Ethiopia.

**Table 4:** Model fitted to the firstborn data with maternal age at childbirth

	Model0_A	Model1_A	Model2_A
*Age at status (event or censor)*			
0	− 3.39 (0.08)***	− 3.30 (0.09)***	− 2.82 (0.52)***
1–5	− 4.19 (0.11)***	− 4.10 (0.12)***	− 3.59 (0.52)***
6–11	− 4.40 (0.14)***	− 4.32 (0.15)***	− 3.81 (0.53)***
12–23	− 4.55 (0.16)***	− 4.47 (0.17)***	− 3.93 (0.54)***
24–59	− 4.64 (0.22)***	− 4.56 (0.22)***	− 4.00 (0.56)***
*Maternal age at child’s birth*			
20–35		− 0.18 (0.11) ~	0.05 (0.11)
35 +		0.17 (0.59)	0.62 (0.60)

Level of significance codes: ***p $<$ 0.001, **p $<$ 0.01, *p $<$ 0.05, ~ p $<$ 0.1

## Discussion and conclusion

### Discussion of the results

Early childhood mortality in Ethiopia has declined substantially in the last two decades. However, Ethiopia is still one of five countries that together account for half of all deaths under age five in the world [2]. To reduce the risk of early childhood deaths, focusing on influential demographic, biological and socioeconomic factors associated with high child mortality in Ethiopia is invaluable [37]. It is asserted that the factors relating to the rate of births need careful emphasis [3]; this study focuses on the effect of these factors, particularly on the three reproductive variables: preceding birth intervals, maternal age, and birth order. Several studies have examined the effect of these factors; however, the challenge is that these factors interact. The gap lies in examining the effects of these factors in combination [3].

In both unadjusted and adjusted models of the main factor analysis, the three factors significantly affect the survival status of under-five in Ethiopia. This result is congruent with other studies [9, 13, 40]. However, other studies conducted in Ethiopia have shown the significance of one factor, with insignificant effects of the other factors [1, 41, 42]. In particular, the influence of birth intervals on the death of under-fives is consistently significant. Even when the effect of the other two factors, maternal age at childbirth and birth order, are controlled for, the effect of birth intervals is consistent with previous studies and consistently reveals the significance of its effect on childhood mortality [8, 43–45]. Furthermore, the results show that the risk decreases continuously as the interval becomes longer. Studies by Rutstein [11, 12] showed similar trends for child mortality, in which longer intervals imply a higher chance of survival, although it indicates a lower chance of survival for neonates and infants. In a study conducted based on the first three EDHSs, the author agrees with this trend that consistently higher birth intervals result in a lower risk of under-five mortality [42].

Furthermore, the results of our study reveal that a short interval of less than 18 months is associated with the highest risk of under-five mortality. Most previous studies are in agreement in concluding that birth intervals of less than 2 years are associated with a higher risk of childhood mortality [1, 11, 12, 15, 42]. Unlike maternal age at birth and birth order, the studies are consistently in agreement on the significance of the effect of the birth interval on mortality, and whether the effect of the other two has been controlled for. On the other hand, maternal age also significantly affects under-five mortality in Ethiopia. The results of the main effects analysis show that births from very young (< 20) maternal-age mothers are associated with the highest risk of child mortality; for older women over 35 + years, the risk is also high relative to the middle maternal age group (20–34 years), even controlling for the effect of other reproductive factors. A U-shaped tendency pattern was observed for maternal age, as noted in several previous studies, although the effect of older maternal age was not statistically significant. A study conducted in Nigeria revealed this trend in child mortality by controlling for important factors without birth order [29]. Other studies also exhibited a U-shaped pattern of the maternal age effect on neonatal, post-neonatal, and child mortality, controlling for the influences of other factors, including birth intervals and birth order [46].

The analysis of the main effect of birth order on under-five mortality has found that birth orders of seven and over (7 +) relative to the second to third (2–3) significantly affect the under-five survival rate. Several studies also assessed the effects of birth order on child health and survival but with mixed findings. Some studies found a significant influence of birth order, controlling for the effects of the other two reproductive variables, birth intervals and maternal age [30]. found a significant effect of birth order on child mortality, while maternal age was not significant, the effect of the preceding birth interval was controlled for [47]. However, other studies found that the effect of birth order on child mortality was not significant [1, 48]. In addition, a study conducted in Ethiopia found that the effect of birth order on child mortality was insignificant but concluded that this was misleading to construe that it has no effect on child survival [42].

From the combined effect of maternal age at childbirth and birth order in controlling the main effect of preceding birth interval, we observed that births with birth order 2–3 and maternal age at childbirth 20–34 years are associated with the best survival status. The higher birth order with young maternal age and lower birth order among older maternal age are risky for under-five childhood mortality. This trend reinforces explanations shared by [33].

Furthermore, controlling for the main effect of birth order, births with a young mother aged below 20 with birth intervals of over four years are the most advantageous. On the other hand, a birth with an older maternal age at childbirth and a very short birth interval are the most disadvantageous. A previous study highlighted the benefit of increasing the maternal age at birth for higher birth order if the birth interval is necessarily short [14].

Finally, the effect of the interaction of a preceding birth interval and birth order while controlling for the main effect of maternal age at childbirth showed that the effect of very short birth intervals worsens in higher birth orders. This trend was also revealed in previous studies [7, 34]. The study of considered the interaction of birth intervals with birth orders by controlling the main effect of maternal age and noted that births with short preceding birth intervals (< 18 months) and high birth orders of six and over were associated with the highest risk of child mortality relative to the safer combination of birth orders 2–5 and a long birth interval [7].

### Limitations of the study

This study has some shortcomings. First, the study doesn’t handle or control the problems related to induced abortion, miscarriage, stillbirth, length of gestation, and prematurity, which can have effects on birth intervals whereas short intervals that are attributed with a higher risk of death might be implicitly the impacts of these factors. Second, this is a cross-sectional study based on retrospective data which might have been affected by problems attributed to data quality such as age misreporting, and the omission of births and deaths. Third, the impacts of HIV/AIDS in children have not been handled in the study; as data are not collected for individual children in DHS data in Ethiopia. However, the prevalence of HIV in Ethiopia is less than 1% and the country could control its impacts. Finally, the study has been based on some assumptions. In the first place, the DHS data was collected only from alive mothers and assume that the child mortality among alive and dead mothers is similar. On the other hand, the study is based on assumptions of the proportionality of the covariates.

## Conclusions

After controlling for the main effect of the other variables, the study found a significant influence of the main effect of the three factors (preceding birth interval, maternal age, and birth order) on under-five mortality. It has been shown that relatively shorter birth intervals, the youngest and oldest maternal age at a child’s birth, and a very high birth order imply a steadily higher risk of death in under-five children in Ethiopia. From interaction analysis, we found that regarding maternal age at a child’s birth, 20–34 years is the safest for births of second to third (2–3) birth order. In terms of the combinations of preceding birth intervals and maternal age at a child’s birth, births of maternal age below 20 with very long birth intervals are the most beneficial. Finally, the results of the analysis of the effect of birth intervals and birth order have uncovered that the effect of a very short birth interval is, relatively, the most severe factor among births with very high birth order.

In conclusion, in addition to the main effect of the three reproductive variables, the combined effects of different levels of these variables determine the survival status of early childhood lives.

## Data Availability

The datasets generated and analyzed for the study are available on the Demographic and Health Survey (DHS) program website (dhsprogram.com).
